# Mapping the AAV Capsid Host Antibody Response toward the Development of Second Generation Gene Delivery Vectors

**DOI:** 10.3389/fimmu.2014.00009

**Published:** 2014-01-30

**Authors:** Yu-Shan Tseng, Mavis Agbandje-McKenna

**Affiliations:** ^1^Department of Biochemistry and Molecular Biology, College of Medicine, University of Florida, Gainesville, FL, USA

**Keywords:** AAV vectors, antibody response, AAV capsid structure, antigenic epitopes, parvoviruses

## Abstract

The recombinant adeno-associated virus (rAAV) gene delivery system is entering a crucial and exciting phase with the promise of more than 20 years of intense research now realized in a number of successful human clinical trials. However, as a natural host to AAV infection, anti-AAV antibodies are prevalent in the human population. For example, ~70% of human sera samples are positive for AAV serotype 2 (AAV2). Furthermore, low levels of pre-existing neutralizing antibodies in the circulation are detrimental to the efficacy of corrective therapeutic AAV gene delivery. A key component to overcoming this obstacle is the identification of regions of the AAV capsid that participate in interactions with host immunity, especially neutralizing antibodies, to be modified for neutralization escape. Three main approaches have been utilized to map antigenic epitopes on AAV capsids. The first is directed evolution in which AAV variants are selected in the presence of monoclonal antibodies (MAbs) or pooled human sera. This results in AAV variants with mutations on important neutralizing epitopes. The second is epitope searching, achieved by peptide scanning, peptide insertion, or site-directed mutagenesis. The third, a structure biology-based approach, utilizes cryo-electron microscopy and image reconstruction of AAV capsids complexed to fragment antibodies, which are generated from MAbs, to directly visualize the epitopes. In this review, the contribution of these three approaches to the current knowledge of AAV epitopes and success in their use to create second generation vectors will be discussed.

## Introduction

Adeno-associated viruses (AAVs) are a promising gene delivery vector system. They are small (~26 nm) non-enveloped viruses belonging to the *Parvoviridae*, are assembled with *T* = 1 icosahedral capsid symmetry, and package a 4.7 kb single-stranded (ss) DNA genome (1). There are over 100 AAV genomic isolates and 13 human and non-human serotypes described. These viruses have different transduction efficiencies in different tissues dictated by the capsid sequence (2). To date, no diseases have been associated with wild-type AAV infection. Further, AAVs can transduce both dividing and non-dividing cells and sustain long-term gene expression in non-dividing cells (3). All these properties make them desirable vectors for therapeutic gene delivery.

Recombinant AAV (rAAV) vectors, used in clinical trials, contain a desired transgene cassette flanked by two inverted terminal repeats (ITRs) instead of the wild-type viral genome flanked by these elements (4). In recent years, the AAV gene delivery system has been successfully utilized in several animal and human clinical trials. In an ongoing hemophilia B trial, therapeutic levels of Factor IX protein has been maintained in patients for over 2 years with only one infusion of an rAAV8 vector packaging this gene (5). In addition, rAAV2 vectors, encoding the retinal pigment epithelium-specific 65 kDa protein, improved vision in Leber’s congenital amaurosis (LCA) patients, without any significant side effects (6–9). rAAV vectors have also been developed for the transduction of a variety of other cells in addition to liver and the eye, including, as examples: brain cells for the treatment of Parkinson’s disease (10) and Canavan disease (11); skeletal muscle for the treatment of emphysema, lipoprotein lipase deficiency, and muscular dystrophy (12–14); and heart muscle for the treatment of heart failure (15). Significantly, in 2012, an AAV1 vector, encoding the lipoprotein lipase, was approved as a gene therapy treatment in Europe (16), heralding a new era for this vector system.

However, despite the above successes, several obstacles must still be overcome for full realization of the AAV vector system in patient care and treatment. One of the most important of these is pre-existing immunity. Serologic studies have shown that the majority of the human population has been exposed to wild-type AAVs (17–19). For example, the prevalence of anti-AAV antibodies in the human population has been reported to be ~40–70%, with the most reactivity against AAV2, the most studied and best characterized of the AAV serotypes. Although rAAV vectors used for gene delivery do not carry viral genes and are unable to drive viral protein synthesis, they are assembled from wild-type viral capsid shells; thus, the host immune response to the vector can be influenced by prior exposure to wild-type AAV. A pre-existing antibody response against AAV can initiate an immune memory response, which could impede gene delivery. For example, neutralization effects from pre-existing antibodies have been reported to decrease transduction efficiency, even at low antibody titers (20–22). For this reason, in the recent gene delivery trial for hemophilia B, individuals with evidence of pre-existing AAV antibody immunity were excluded from participation (5). Thus, to develop the AAV vector system into a more practical and efficacious gene transfer system, it is important to understand how antibodies interact with the AAV capsid, especially to map dominant epitopes, both neutralizing and non-neutralizing. With sufficient information on the AAV antigenic structure, combined with data on capsid determinants of tissue tropism and transduction, it would be feasible to design a neutralization-escaping vector, which can evade the host antibody immune response while retaining desired tissue tropism and transduction efficiency.

Here we will briefly review the AAV capsid structure and what is known about the effects of its interaction with antibodies, and then discuss example approaches utilized for mapping capsid antigenic epitopes. The three most common approaches include: directed evolution, an indirect method for obtaining the antigenic information through the selection of AAV variants under antibody pressure; epitope searching, which utilizes peptide scanning, peptide insertion, or site-directed mutagenesis; structural biology, namely cryo-electron microscopy and three-dimensional (3D) image reconstruction (cryo-reconstruction), which directly visualizes the antigenic sites on the capsid by 3D reconstruction of capsid-fragment antigen binding (Fab) complexes.

## AAV Capsid Structure

**Figure 1:**
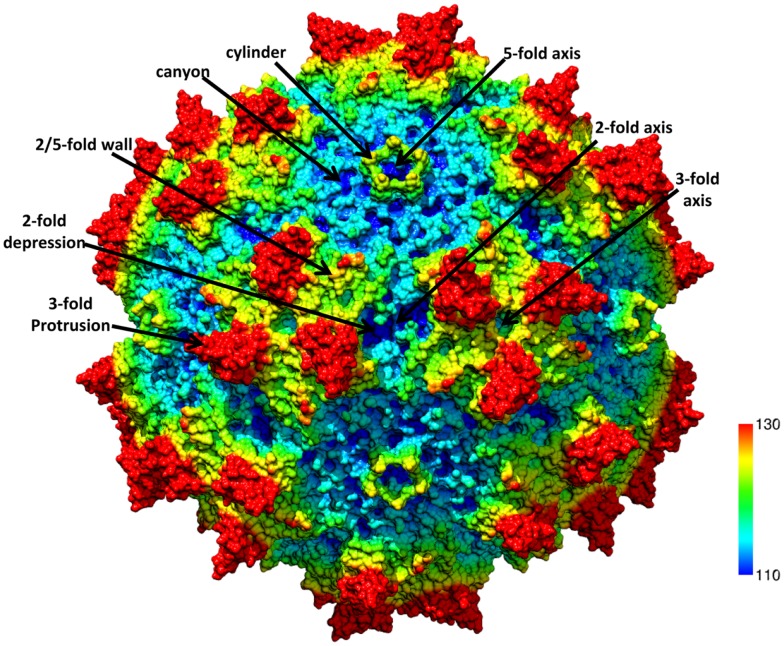
**The AAV capsid**. Radially color-cued (from capsid center to surface: blue-green-yellow-red; ~110–130 Å) of the AAV1 capsid generated from 60 VP monomers (RCSB PDB # 3NG9). The approximate icosahedral 2-, 3-, and 5-fold symmetry axes are as well as the AAV capsid surface features are indicated by the arrows and labeled. This image was generated using the Chimera program (40).

**Figure 2:**
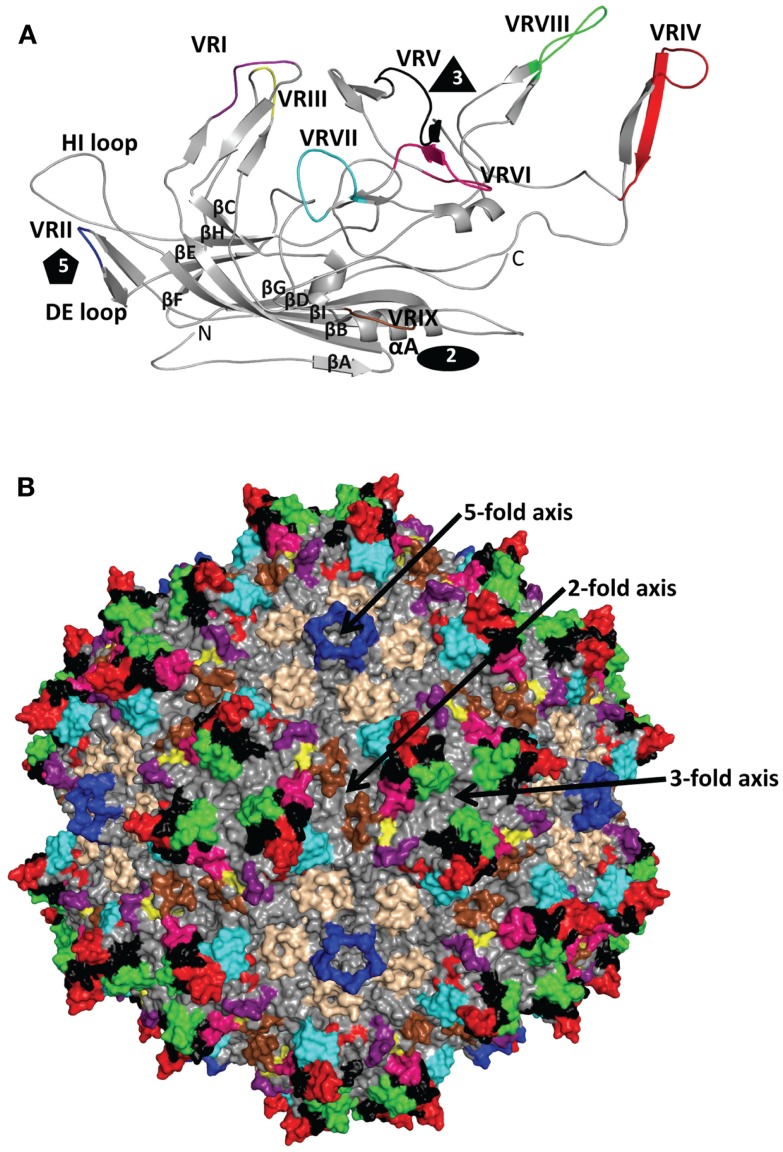
**AAV variable regions**. **(A)** A ribbon diagram representation of the ordered overlapping VP3 monomer region of AAV1. The conserved β-barrel core motif (βBIDG-βCHEF, gray), conserved αA helix, DE loop (between βD and βE), HI loop (between βH and βI), VR-I to VR-IX [defined (26)] are colored; I: purple, II: blue, III: yellow, IV: red, V: black, VI: hot pink, VII: cyan, VIII: green, and IX: brown; and labeled. The approximate positions of the 2-, 3-, and 5-fold axes are indicated by the filled oval, triangle, and pentagon, respectively. The N and C labels are the N- and C-terminal ends of the ordered VP region, respectively. **(B)** The capsid surface of AAV2 with VR-I to VR-IX colored as in **(A)**. The approximate icosahedral 2-, 3-, and 5-fold symmetry axes are indicated and labeled as in Figure 1. Both **(A)** and **(B)** were generated with the PyMOL program (http://www.pymol.org).

The ~4.7 kb genome of the AAVs contains three open reading frames (ORFs), *rep*, *cap*, and *aap*, flanked by two ITRs (~145 kb). This genome is packaged into *T* = 1 icosahedral capsids that are ~26 nm in diameter. The *cap* ORF encodes three overlapping structural capsid viral proteins (VPs): VP1, VP2, and VP3, in a ratio of 1:1:10, which assemble the capsid. A total of 60 VPs assemble the capsid by 2-, 3-, and 5-fold symmetry-related interactions (Figure 1). The VP3, the major capsid component, is able to assemble the capsid as long as the assembly activating protein (AAP) encoded by the *aap* ORF, is present (23, 24). Three-dimensional structures have been determined for AAV1-AAV9, the clade and clonal isolate representatives of the over 100 genomic sequences known for the human and non-human primate AAVs (2), by either X-ray crystallography and/or cryo-reconstruction (25–32). In all these structures only the VP3 common sequence is ordered. While the AAVs have a sequence similarity that ranges from ~55 to 99%, they are structurally very similar (33). The VP topology consists of a conserved alpha helix (αA) and a eight stranded anti-parallel (βB-βI) β-barrel core with large inter-strand loops (Figure 2A) that form the exterior surface of the capsids. A comparison of the AAV2 and AAV4 structures, two of the most distantly related, identified nine common variable regions (VRs), designated VR-I to VR-IX (Figures 2A,B), located on the capsid surface at the top of the inter-strand loops (26, 34). The AAV capsid surface is characterized by depressions at the 2-fold axes (dimple), surrounding a cylindrical channel at the 5-fold axes (canyon), and protrusions surrounding the 3-fold axes (Figure 1). A wall or plateau is located between the depression at the 2-fold axis and surrounding the 5-fold channel, the “2/5-fold wall” (Figure 1) (35). The VRs contribute to local topological differences between the AAV capsid surfaces. For example, VR-II forms the top of the 5-fold channel; VR-IV, V, and VIII form the top of the 3-fold protrusion and VR-VI and VR-VII form their base; and VR-I, III, VII, and IX contribute to the 2/5-fold wall (Figure 2B). The VRs also dictate functional differences, including receptor attachment, transduction efficiency, and antigenic reactivity between the AAVs (26, 28, 30, 36–39).

## AAV and Antibodies

All viral vectors are susceptible to the immune response from the host (41). The most detrimental immune threat that AAV vectors encounter soon after administration is the B-cell mediated antibody response (42, 43). Antibodies use their complementarity determining region (CDR), located on the end of Fab region, to interact with antigens by specific surface complementarities (44). This binding site, or “epitope,” is generally located on the capsid surface of viruses. Antibodies against viruses may neutralize infectivity prior to viral attachment to host cell receptors, or post attachment; interfering with internalization or fusion at the cell surface, or during endosomal trafficking (45–48). Other antibody neutralization mechanisms include antibody-mediated phagocytosis, complement binding and activation, opsonization, and antibody-dependent cellular cytotoxicity (ADCC) of infected cells (49–52). As already stated above, in humans the prevalence of AAV antibodies in healthy individuals is high and ranges from ~40–70% depending on serotype. Previous reports indicate that the highest prevalence of anti-AAV immunoglobulin G antibodies in humans was for AAV2 (~60–70%) and AAV1 (~35–70%), followed by AAV9 (~50%), AAV6 (~50%), AAV5 (~40%), and AAV8 (~40%) [e.g., Ref. (17, 53)]. For the AAVs, neutralization mechanisms have been described for only three monoclonal antibodies (MAbs), A20 and C37-B against AAV2, which act at the post-entry steps and receptor attachment, respectively (54, 55), and ADK8 against AAV8, which acts at a post cell/pre-nuclear entry step (37).

Given the reported detrimental effects of anti-AAV antibodies on transgene expression, it is not surprising that the most successful applications of the AAV vector system have been in the eye and brain, immuno-privileged sites when vector is directly injected, or in patients with low or no anti-AAV antibodies titers prior to vector administration. Furthermore, since the AAV capsid structures are similar, different anti-AAV antibodies may cross-react, as has been observed, for example, between AAV2 and AAV3 that are highly similar at the sequence level (55), and recently for AAV1 and AAV5 which are disparate in at the sequence level (56). Boutin et al. (17) showed that patients with a positive AAV2 serum response were also seropositive for AAV1, AAV5, AAV6, AAV8, and AAV9 in 93, 52, 59, 57, and 58%, respectively, of the samples tested. Thus a new generation of AAV vectors is needed to circumvent the neutralization effects from pre-existing antibodies. The development of these new vectors will largely depend on available AAV antigenic and structure information. To obtain the antigenic information, three main approaches have been used. These are discussed below.

## Directed Evolution

Without knowledge of AAV capsid biology, structure, or immunogenic sites, directed evolution serves as a strategy to generate neutralization-escaping AAV variants. It is a high-throughput molecular engineering procedure, which mimics natural evolution through iterations of genetic diversification under artificial selection pressure (57). For generating neutralization-escaping AAV variants, wild-type *cap* genes, from one or several AAV serotypes, are mutated to generate a large genetic plasmid library, which can generate numerous capsid variants through recombination during plasmid transfection. During viral infection with viruses arising from the recombination process, a selective pressure, in this case antibodies, is applied. Only the variants that can circumvent the antibody barrier presented to infect the desired cells will transduce and drive progeny virus synthesis. The successful variants are then recovered and amplified for the next round of selection. After several cycles of selection, additional mutagenesis can also be introduced before further selection to increase viral fitness (58). Comparison of the capsid sequences of the final resulting variants and the wild-type input viruses provides the antigenic information and the effect of the changes on tropism and transduction efficiency. Generally, there are two strategies to create the genetically diverse library, one is using error-prone mutagenesis to randomize the capsid DNA sequences; the other is to shuffle the DNA sequences of different AAV serotypes.

### Error-prone mutagenesis

By a “sloppy” polymerase chain reaction (PCR), random point mutations can be introduced into an ORF at a certain rate. Tuning the PCR conditions can also introduce a different number of mutations into the target gene sequence (59). Perabo et al. (60) used this approach to generate an AAV2 *cap* mutant library for directed evolution under human sera selection. Approximately, 70% of the neutralization-escaping variants obtained contained point mutations clustered on the external face of the capsid at the 3-fold protrusion (Figure 1). The two most frequently selected mutations were amino acids 459 and 551 (Table 1) located in AAV VR-IV and VR-VII, respectively (Figure 2). The variant with the best neutralization escape capability carried the double mutation R459K/N551D. The single (R459G or N551D) and the double (R459K/N551D) mutant variants had comparable genome packaging, infectivity, and particle titers to wild-type virus. However, the N_50_ value, the amount of serum required to decrease transduction by 50%, for R459G, N551D, and R459K/N551D were 4.1-, 3.3-, and 5.5-fold higher, respectively, than the corresponding N_50_ value obtained for the wild-type AAV2.

**Table 1:** **Antigenic sites identified by polyclonal antibodies**.

Antibody sample	Method	Residues	Reference
Rabbit anti-AAV2 serum	Directed evolution	12, 42, 117, 152, 180, 258, 418, 493, 567, 587, 713, 716	Maheshri et al. (61)
Human serum	Directed evolution	459, 551	Perabo et al. (60)
Human serum	Peptide scanning	17–28, 113–124, 241–260, 305–356, 401–420, 443–460, 473–484, 697–716	Moskalenko et al. (62)
Human serum	Peptide insertion	534, 573, 587	Huttner et al. (63)
Human serum	Site-directed mutagenesis	471, 497, 498, 531, 548, 550, 586, 587, 705, 708	Lochrie et al. (38)
Human IVIG	Site-directed mutagenesis	264, 265, 269, 471, 491, 497, 498, 502, 527, 531, 532, 544, 550, 574, 586, 705, 706, 708	Lochrie et al. (38)

Maheshri et al. (61) improved this strategy by combining the error-prone PCR with a staggered extension process, which utilized a short time polymerase-catalyzed extension (64) to produce AAV2 variants. Mutant capsids were next selected for infectivity in HEK293 cells in the presence of a neutralizing rabbit anti-AAV2 serum. Nine mutants, which contained different combinations of mutations at amino acid positions 12, 42, 117, 152, 180, 258, 418, 493, 567, 587, 713, or 716, had neutralizing titers that were 3-fold higher than the wild-type virus (Table 1) (61). All the successful variants carried a T716A mutation. This residue is located on the capsid surface at the 2/5-fold wall next to VR-IX (Figures 1 and 2B). One variant, r2.15, had a 96-fold improvement in transduction compared to wild-type AAV2 and the ability to mediate moderate gene delivery at a low 1:2 serum dilution. This variant has two mutations not present in the others: T567S and N587I. T567S is in a minor epitope of the A20 MAb previously identified by pepscan and peptide competition (55). Residue 587 is in VR-VIII (Figure 2) and it is located proximal to residues involved in heparan sulfate proteoglycan (HSPG) receptor binding in AAV2 (65, 66).

Maersch and colleagues (67) also used error-prone PCR to establish an AAV2 *cap* library with the mutations focused only on amino acids located in the 3-fold region previously identified as being immunogenic: 449, 458, 459, and 551 (60), and 493 (61). The directed evolution was carried in HEK293 cells under the selection of neutralizing human serum. The resistance to neutralization of six resulting variants was compared to those of wild-type AAV1, AAV2, and the double mutant R459K/N551D generated by Perabo et al. (60). Two of the variants, with substitutions at 459, 493, and 551, outperformed the best variants from the previous selections by Maheshri et al. and Perabo et al. (60, 61). This observation indicated that fine tuning single amino acid types on the capsid surface can dramatically change the immunogenicity of an epitope. Residue 493 is located in VR-V, which as previously mentioned, together with VR-IV and VR-VIII form the top of the protrusions surrounding the 3-fold axis. Thus the data from error-prone mutagenesis provides indications that the 3-fold region is immuno-dominant in the AAVs.

### DNA shuffling

DNAse digestion followed by polymerase ligation can generate a chimeric capsid variant from different AAV serotypes. Grimm et al. (68) applied this method to randomly combine the cap sequences from AAV2, AAV5, AAV8, AAV9, caprine AAV, avain AAV, and bovine AAV prior to selection. They first selected capsid variants capable of human liver cell transduction and then selected in the presence of pooled human anti-sera (Intravenous immunoglobulin, IVIG). This strategy created a single chimera named AAV-DJ. The amino acid sequence for AAV-DJ was closely related to AAV2, AAV8, and AAV9 at 92, 88, and 85% identity, respectively. Interestingly, the IVIG selection generated this variant with higher homology to AAV8 compared to variants selected for human liver cell transduction alone, which were mostly similar to AAV2. This implies that the IVIG selection pressure eliminated variants with AAV2 epitope(s) from the resulting clones, consistent with the higher percentage of anti-AAV2 sero-prevalence in the human population compared to other AAV serotypes. AAV-DJ was capable of transducing mice passively infused with IVIG prior to infection at low (4 mg) IVIG dose to similar levels as parental AAV8 and AAV9 while AAV2 transduction was abolished in either high (20 mg) or low (4 mg) IVIG dose. The AAV-DJ was also inhibited at the high IVIG dose.

Another group, Koerber et al. (69), also generated chimeric AAV capsids through DNAse I digestion and polymerase ligation which displayed neutralization escape capability. The library was established from the *cap* ORF of AAV1, AAV2, AAV4, AAV5, AAV6, AAV8, and AAV9. The selection was carried once in HEK293 cells to optimize just for infectivity without any antibody pressure. However, surprisingly, some IVIG neutralization-escaping variants were generated. Four of the final seven chimeric capsids obtained showed improved IVIG neutralization resistance compared to the parental serotypes despite ~90% sequence similarity. Three of the variants that had higher resistance than the parental AAV2 had a ~80 aa stretch from AAV9 or a V709I mutation and the last C-terminal 19 aa from AAV6. The C-terminal stretch contained a previously described AAV2 epitope, 697–716 (AAV2 numbering, see below) (62). One of the chimeras, cB4, was similar to AAV1/6 at the sequence level, and had >400-, 8-, and 2-fold resistance to IVIG neutralization compared to AAV2, AAV1, and AAV6, respectively. While it was difficult to define which region of this chimera dictated its strong immune evading ability, it contained a Y706H substitution. Both the Y706H and V709I changes would be located in the 2/5-fold wall.

Other examples of chimeras selected for infectivity without antibody selection which display increased antibody neutralization resistance have been reported. Li et al. (70) used iterative cycles of infection to select variants with hamster melanoma cell tropism started from a shuffled library constructed from AAV serotypes 1–9, except serotype 7. In this study, a specific chimeric AAV variant was isolated, containing residues 1–409 from AAV1, 410–450 from AAV8, 451–704 from AAV2, and 705–736 from AAV9. This chimera, chimeric-1829, did not show any cross-reactivity to anti-sera from mice immunized with AAV1, AAV8, and AAV9. The sera from mice immunized with AAV2 had some cross-reactivity to chimeric-1829, but the neutralizing antibody (Nab) titer was 25-fold lower than the titer for wild-type AAV2 (70). In another study, Yang et al. (71) used DNA shuffling and *in vivo* selection to generate a muscle-cell targeting AAV chimera from serotypes 1–9 with a different antigenic reactivity compared to the parental serotypes. The mutant, M41, assembled from AAV1, AAV6, AAV7, and AAV8 sequences, showed a higher resistance to IVIG neutralization compared to AAV2. At a 1:64 dilution of IVIG, AAV2 infectivity decreased to ~33%, whereas M41 infectivity still remained at 83% compared to controls without IVIG incubation (71). The wild-type AAV8 infectivity remained at ~94% under these conditions, suggesting that the sequence of M41 derived from AAV1, AAV6, and AAV7, not those from AAV8, resulted in the increased susceptibility to IVIG recognition. This observation is consistent with the fact that residues 410–450 contributed to M41 by AAV8 are mostly internal in the capsid and form core β-strand regions of the VP structure.

## Epitope Searching

Rather than viral evolution in the presence of antibodies to obtain information on the antigenic regions of the capsid, epitope searching focuses directly on the interaction(s) between antibodies and peptides generated from the capsid protein sequence to map possible epitopes. This approach utilizes three main strategies, peptide scanning, peptide insertion, and site-directed mutagenesis.

### Peptide scanning

Moskalenko et al. (62) scanned the entire AAV2 capsid amino acid sequence for potential epitopes using a total of 91 15-mer peptides that overlapped by five amino acids. The peptides were tested for their ability to inhibit capsid binding by AAV2 neutralizing human serum samples in an ELISA assay in which the peptides were applied to an AAV2 capsid coated ELISA plate. This study identified several overlapping peptides regions, two within the VP1u sequence, 17–28 and 113–124, and six within the common VP3 sequence, 241–260, 305–356, 401–420, 443–460, 473–484, and 697–716 (Table 1) (62). As already stated, there is no structure available for the VP1u. The VP3 sequences are localized to βB, strands βD-βE and the intervening loop that forms the 5-fold channel including VR-II, strands βF-βG, the outer finger of the 3-fold protrusion that contains VR-IV, a strand region of the GH loop, and the 2/5-fold wall that contains VR-IX, respectively, in the VP3 crystal structure. The peptides containing residues 305–356, 401–420, 443–460 were considered the core neutralizing peptides. A number of these peptides are located in VP regions that are inside the capsids, and thus their ability to block capsid binding by human serum likely reflects the polyclonal nature of the human serum, which must include antibody responses to denatured VP regions or fragments.

Wobus et al. (55) also used a peptide mapping strategy to determine the epitopes of three mouse MAbs, A1, A69, and B1 previously reported to react against AAV2 (72, 73). An AAV2 *cap* gene fragment phage display library was screened with the antibodies and positive clones were scanned for binding by the antibodies, as overlapping 15-mer peptides on a membrane, and further confirmed by peptide competition in a Western blot. Linear epitopes were mapped for A1 (residues 123–131) within VP1u, A69 (residues 171–182) in the VP1/VP2 region and for B1 (residues 726–733) at the C-terminus of VP3. Consistently, these antibodies react against denatured capsids. The epitopes for three conformational AAV2 directed antibodies, A20, C37-B, and D3 (55) were also identified through a similar strategy. Overlapping 10-mer peptides (covering the AAV2 capsid sequence) detected by the antibodies were confirmed by an ELISA assay. Multiple peptides were identified for these antibodies consistent with their recognition of the assembled capsid, although C37-B and D3 were also capable of recognizing the VPs (55). The epitopes proposed for the three MAbs were: 272–281, 369–378, and 566–575 for A20; 492–503 and 601–610 for C37–B; and 474–483 for D3 (Table 2). The A20 epitope residues are mostly located below surface loops containing VR-I (for 272–281), the HI loop (for 369–378), and VR-V (for 566–575); the C37-B epitope peptides are located in VR-V (492–503) and buried at the 3-fold axis (601–610), and the D3 epitope is buried close to the 3-fold axis (474–483) (Figure 2B). As previously mentioned, the A20 MAb neutralizes virus infection at a post cell entry step and the C37-B antibody inhibits HSPG receptor attachment by AAV2. The D3 antibody is non-neutralizing, which is consistent with the mostly buried location of its mapped epitope.

**Table 2:** **Antigenic epitopes identified using monoclonal antibodies**.

AAV	MAb	Method	Residues						Reference
			200–299	300–399	400–499	500–599	600–699	700–731	
AAV1	4E4	Cryo-EM			456–459, 492–498				Gurda et al. (36)
AAV1	5H7	Cryo-EM			494, 496–499	582, 583, 588–591, 593–595, 597			Gurda et al. (36)
AAV2	A20	Cryo-EM	253, 254, 258, 261, 262, 264	384, 385		548, 556	658–660	708, 717	McCraw et al. (39)
		Peptide scanning	272–281	369–378		560–573			Wobus et al. (55)
		Peptide insertion	261	381		534, 573			(55, 63)
		Site-direct mutagenesis	263, 264	384, 385		548		708	Lochrie et al. (38)
	C37-B	Cryo-EM			492–498,	585–589			Gurda et al. (36)
		Peptide scanning			493–499	500–502	601–610		Wobus et al. (55)
		Peptide insertion				534, 573, 587			(55, 63)
	D3	Peptide scanning			474–483				Wobus et al. (55)
		Peptide insertion	261	381		534, 573			Wobus et al. (55)
AAV5	3C5 site A	Cryo-EM	254–261	374, 375	483, 485–492, 494, 496, 499	500, 501			Gurda et al. (36)
	3C5 site B	Cryo-EM	246			530, 532–538	653, 654, 656, 657	704–708	Gurda et al. (36)
AAV8	ADK8	Cryo-EM				586–591			Gurda et al. (37)

### Peptide insertion

Wobus et al. (55) attempted to further confirm the binding sites for the A20, C37-B, and D3 antibodies as well as another antibody named C24-B using mutant AAV2 capsids onto which a 14 amino acid integrin binding ligand, L14, had been inserted. Prior to the availability of the AAV2 capsid structure, Girod et al. (74) utilized the 3D structure of canine parvovirus (75) to generate a 3D homology model for AAV2, which was used for predicting potential capsid surface sites onto which this peptide could be inserted for re-targeting AAV2 (74). Six such sites, 261, 381, 447, 534, 573, and 587, were identified. Testing of the antibody binding properties of these insertion mutants with the A20, C37-B, D3, and C24-B antibodies showed that the insertions at 261, 381, 534, and 573 decreased A20 binding; at 534, 573, and 587 decreased C37-B binding; at 261, 381, 534, and 573 decreased D3 binding; and at 534, 573, and 587 decreased C24-B binding (Table 2). These insertion points did not overlap with the epitopes predicted based on peptide scanning data, except for residue 573 for A20 (55). For the A20 and C37-B MAbs, a number of the sites do overlap with or are close to epitope regions mapped using site-directed mutagenesis and structural biology as discussed below.

Huttner et al. tested the ability of these six insertion mutants to evade binding by human serum samples in an ELISA assay (63). The A20 and C37-B antibodies were used as positive controls. Insertion mutations at amino acid positions 534 and 573 reduced human anti-sera binding, in 19/29 tested samples, by up to 30% compared to the wild-type AAV2. Both of these residues are buried inside the protrusions surround the 3-fold axis. Thus their negative impact on serum binding is likely due to disruption of the surface loops that assembles the protrusions suggested above to be important for antigenic reactivity for the AAVs. The mutant with an insertion at position 587 only slightly impaired serum binding in ELISA assays, but was able to transduce Hela cells in the presence of human serum. The inserted L14 ligand also enabled this mutant to infect B16F10 cells, a cell line which is non-permissive to the parental AAV2, in the presence of human serum. These observations are consistent with the fact that residue 587 of AAV2 is located in a capsid region involved in immunogenicity, e.g., the C37-B epitope, and cellular attachment, e.g., proximity to the HSPG binding site (65, 66).

### Site-directed mutagenesis

The best example of the use of site-directed mutagenesis for antigenic epitope mapping is provided by the study by Lochrie et al. (38). This group used the crystal structure of AAV2 (32) to identify sites for mutations on the capsid surface at amino acid positions predicted to be potential antigenic sites based on the docking of a murine IgG2a. All the mutants (57 alanine substitutions, 41 non-alanine substitutions) were screened for binding and neutralization ability with A20, three individual human serum samples, and IVIG. The mutated positions that decreased neutralization by A20 were 263, 264, 384, 385, 548, and 708. These residues are different to the amino acid stretches mapped by Wobus et al. (55) and Hunter et al. (63), but are located structurally proximate to these residues and are all clustered on the 2/5-fold wall (Table 2 and Figure 1). The three human sera and IVIG screening identified several epitopes (Table 1) due to the complexity of the polyclonal response being tested. Different mutants showed resistance to different serum, and the mutations that resulted in neutralization escape from the individual serum and IVIG were located over a capsid region that was three times larger than an average Fab epitope footprint and spanned the 3-fold protrusions as well as the 2/5-fold wall. In addition, the ability to escape from human sera and IVIG differed for some mutants. For example, mutant R471A and mutant N587A were both resistant to all three tested human sera, but mutant N587A was not resistant to IVIG neutralization. Significantly, two A20 neutralization escape mutants, E548A and V708A, also escaped neutralization by these sera, showing similarity in the murine and human immune response to AAV2. In addition, AAV2 V708 is positionally equivalent to or proximal to the V709 position in the chimera generated by Koerber et al. which when mutated to an isoleucine (V709I) improves resistance to IVIG neutralization (69).

## Structure-Based Approach

Cryo-reconstruction is a powerful technique for studying the structures of macromolecular complexes, including viruses and their complexes with receptors and antibodies. This method has thus been applied for the study of AAV capsids bound to Fabs, generated from MAbs, toward 3D characterization of the AAV capsid antigenic structure. Purified Fabs and AAV samples are mixed and incubated prior to vitrification on an electron microscope grid. For cryo-reconstruction, a large number of 2D projections of the sample vitrified in native state, at different orientations, are combined and processed to generate a 3D image reconstruction. Current resolutions for AAV:Fab complex structures range from subnanometer to ~20 Å. For identification of the antibody footprint, available atomic structures for the AAV capsids (35) and homology models for the Fabs are fitted into the reconstructed density map, in an approach termed pseudo-atomic model building, to provide the information on interacting sites. Below we review the current AAV:Fab complexes and the antigenic sites arising from these studies.

### Cryo-reconstruction of AAV1/6:Fab complexes

AAV1 and AAV6 differ by 6/736 VP1 residues with 5/6 of them located within the VP3 common region, are cross-reactive, and belong to the same antigenic clade A (2), yet they differ in their cellular tropisms pointing to a key role for the specific amino acid differences in dictating these properties. The muscle tropism of AAV1 has made it an attractive vector for several gene delivery applications [see clinicaltrials.gov and (76)] and like AAV2, epidemiological studies show a high level of pre-existing anti-AAV1 immune response in the general population (17, 53). Efforts to structurally map the antigenic structure of AAV1 have included the cryo-reconstruction of AAV1 complexed with Fabs from two neutralizing mouse MAbs, AA4E4.G7 (4E4) and AA5H7.D11 (5H7) (36, 56). These structures were determined to ~12 and ~23 Å resolution, respectively. The proposed epitopes for the Fabs are residues 456–459 (in VR-IV) and 492–498 (in VR-V) for 4E4 and residues 494, 496–499 (on VR-V) and 582, 583, 588–595, and 597 (on VR-VIII) for 5H7 (Table 2). A structure of AAV6 complexed with the 5H7 Fab, determined to ~15 Å resolution, identified a similar footprint on the capsid. These epitopes are located on the 3-fold protrusions assembled from VR-IV and VR-VIII from one VP monomer and VRV from a neighboring VP. The binding of the Fabs occur in different orientations. 4E4 binds to the “outer side” of the protrusion, with the long axis of Fab toward and across the 2-fold axis. Steric hindrance limits the binding of this Fab to just one at a time across the 2-fold axis, thus the occupancy is 0.5. 5H7 binds on the “inward facing side” of the 3-fold protrusion with its density centered at the 3-fold axis; hence, on average, only one Fab can bind to a group of three protrusions, resulting in occupancy of 0.3. Binding and transduction studies suggested that these two antibodies neutralize infection by either competing with cell surface receptor attachment or inhibition of a step post cellular entry (56).

### Cryo-reconstruction of AAV2:Fab complexes

For AAV2, which has broad tissue tropism and has been the vector most often used for clinical gene delivery applications (76), the binding sites for the A20 and C37-B antibodies have also been mapped by cryo-reconstruction (36, 39). These structures were determined to 8.5 and ~11 Å resolution, respectively. The A20 footprint, determined by several approaches including pseudo-atomic model building, includes residues 253, 254, 258, 261–264, 384, 385, 548, 556, 658–660, 708, and 717 (Table 2) (39). These residues had some overlap with those previously described based on peptide insertion as well as site-directed mutagenesis (see above) but extended the footprint to include additional residues (Table 2). As stated above, when discussing the A20 epitope mapped by site-directed mutagenesis, there was no overlap to the footprint predicted by peptide scanning, but the residues are within the same capsid region. Significantly, the binding site included residue contributions from symmetry-related VP monomers, confirming the conformational nature of the A20 epitope. The residues are located in AAV VR-I and VR-III and the HI loop from one VP monomer, and VR-VII and VR-IX from a second VP monomer. The AAV2 C37-B footprint, based on a pseudo-atomic model built into the cryo-reconstructed density map using the AAV2 crystal structure and the structure of a generic Fab, includes residues 492–498 (on VR-V) from one VP monomer and 585–589 (on VR-VIII) from another VP monomer (Table 2) (36). As already stated above, this region of the 3-fold protrusion overlaps with the AAV2 HSPG binding site. This epitope overlaps with binding residues determined based on peptide scanning and insertion (Table 2).

### Cryo-reconstruction of AAV5:Fab complexes

AAV5 is one of the most divergent AAV serotypes with respect to sequence and structure and is classified as a clonal isolate based on antigenic non-cross-reactivity with other AAVs. As with the other AAVs, efforts are underway to characterize its antigenic structure because human sera also show pre-existing reactivity. While a number of mouse MAbs have now been generated against the AAV5 capsid (56, 77), an antigenic footprint has only been characterized for one, BB3C5.F4 (3C5), based on a structure reconstructed to ~16 Å resolution (36). The 3C5 MAb is non-neutralizing, and the observation that both the variable and constant regions of its Fab contact the capsid was suggested as being possibly due to it being an affinity immature antibody (36). This is because the MAb was generated only 4 days after AAV5 capsid immunization of a mouse that had been previously immunized with AAV1 (56). The Fab density covered the majority of the capsid surface, with the exception of the 3-fold axis. Pseudo-atomic model building into the reconstructed complex density identified two contact regions designated site A and site B with the variable region being better accommodated in site B. This site B region extended from the 2/5-fold wall toward the 5-fold axis and included residues 246, 530, 532–538, 651, 653, 654, 656, 657, 704–708 (Table 2). These residues are structurally located in VR-I, VR-VII, the HI loop, and VR-IX. Interesting, the site B footprint is similar to that mapped for the A20 MAb against AAV2 (Table 2) (38, 39, 55), despite AAV2 and AAV5 being highly antigenically divergent (2).

### Cryo-reconstruction of AAV8:Fab complexes

AAV8 has shown great promise as a liver tropic vector and is currently being utilized as the gene delivery vector in a clinical trial for hemophilia B (5). While the patients selected to receive the Factor IX gene had little or no pre-existing antibody response against AAV8, these patients have since developed an antibody response and as such a repeat administration would require the use of an alternative serotype or AAV8 variant with altered antigenic reactivity. The only information on the antigenic structure of AAV8 has been provided by a cryo-construction of this serotype complexed with a neutralizing antibody, ADK8 (23, 37). This structure was determined to 18.7 Å resolution (37). The AAV8 crystal structure and that of a generic Fab were docked into the reconstructed density to create a pseudo-atomic model of the complex. The footprint predicted from this model was confirmed by mutagenesis, biochemical, and *in vitro* assays to be residues 586–591 located in VR-VIII. This region is located on the inner face of the protrusions facing the 3-fold axis. The mechanism of neutralization by the ADK8 antibody is currently unknown but occurs post cellular attachment and pre-nuclear entry (37). Significantly, an AAV8 vector mutated at residues 589–591 is capable of evading neutralization by ADK8 and retains the liver transduction efficiency of the parental AAV8 vector in a mouse model (78). This study thus provides a proof of the concept that AAV antibody binding sites can be engineered to evade recognition while retaining the natural parental transduction properties.

## Commonalities in Mapped AAV Antigenic Epitopes

**Figure 3:**
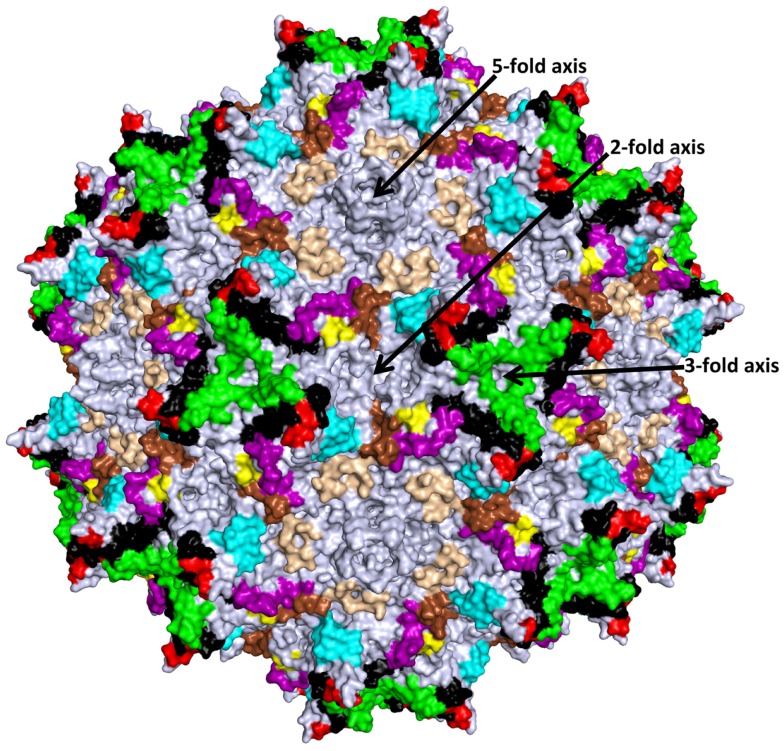
**Structurally mapped AAV antigenic epitopes**. The epitopes identified on the AAV capsid surface by cryo-reconstruction structure are depicted in the colors used for the VRs in Figure 2 based on overlap with the VR amino acids; aa253–271: purple; aa383–386: yellow; aa456–459: red; aa492–515: black; aa544–557: cyan; aa582–597: green; aa659–669: wheat; and aa709–720: brown. Amino acids 659–669 (wheat) were not previously described as VR regions. The approximate icosahedral 2-, 3-, and 5-fold symmetry axes are indicated and labeled as in Figure 1. This image was generated with the PyMOL program (http://www.pymol.org).

The AAV antigenic epitopes mapped by the structural-based approach show significant overlap among the AAVs despite differences in the amino acid types at these epitopes (Table 2). The 4E4 and 5H7 antibodies against AAV1 and C37-B against AAV2 have epitopes that contain residues in the 492–499 peptide stretch while epitopes for C37-B against AAV2 and ADK8 against AAV8 contain the 585–589 sequence stretch. As already mentioned above, these residues are localized to the protrusions that surround the 3-fold axis (Figure 3). The epitopes of A20 and 3C5, which cover the 2/5-fold wall and the floor of the depression, surround the 5-fold axis and share residues 254, 258, 261, and 708. With the exception of the HI loop structure, which is conserved in all AAV structures so far determined, the AAV antigenic epitopes mapped by cryo-reconstruction are localized to VRs on the AAV capsid surface (Figure 3) (26). This clustering of epitopes suggests a limited number of common antigenic regions on the AAV capsid surface. Significantly, the 3-fold and 2/5-fold regions have been implicated in antibody binding and neutralization for other parvovirus capsids including *Aleutian Mink Disease Virus*, *Human Parvovirus B19*, *Canine Parvovirus*, and *Feline Panleukopenia Virus* (79–83). These observations suggest a commonality in development of the host humoral response against parvovirus capsids, which can inform on the antigenic regions of the AAVs. Thus the epitopes presented here likely represent the capsid sites that dominate antibody recognition by the AAV capsid.

While single mouse MAb footprints, as mapped by cryo-reconstruction or peptide scanning/insertion may not be enough to predict the antigenic repertoire of the polyclonal antibody response present in human sera, the combined information from several AAV:Fab complexes is proving to be predictive of the antigenic structure of these viruses against the human immune response. A comparison of the AAV MAb footprints identified by cryo-reconstruction or for AAV2 also using peptide scanning, peptide insertion or site-directed mutagenesis with the list of antigenic sites obtained from screening capsid mutants against human sera and IVIGs neutralization shows significant overlap (Tables 1 and 2). For example, AAV2 residues 497, 498, 586, and 587, which when mutated enable AAV2 to escape from serum and IVIG neutralization (Table 1) (38), are part of the C37-B MAb epitope identified cryo-reconstruction (36) and peptide insertion (63). The A20 MAb against AAV2, which had a expansive footprint, included escape mutant residues also identified by directed evolution against rabbit serum and the screening of site-directed mutants with human IVIG (Tables 1 and 2). The commonality between AAV2 antigenic footprints and those for the other AAVs, as mapped by cryo-reconstruction, for example C37-B with 4E4 and 5H7 against AAV1/6, C37-B with ADK8 against AAV8, and A20 with 3C5 against AAV5, suggests that a similar overlap will exist between the footprints of these MAbs and the human polyclonal response to their capsid.

## Pros and Cons of Antigenic Mapping Approaches

The approaches discussed above have their own pros and cons. Directed evolution, in the presence of human sera or IVIG, has the potential to generate a selected AAV variant with the ability to escape neutralization and retain its genome packaging capacity and infectivity. In addition, a specific/desired tissue tropism can be selected (70, 71, 84). However, this process is time consuming, and unlike the rational approaches, the outcome is much harder to predict. Peptide scanning and the structural-based approaches focus on identifying epitopes first and then designing vectors to escape antibody binding. Peptide scanning has the potential to detect interactions from serum or IVIG which resemble the situation in the natural host. However, the epitopes detected are mostly linear and may not represent the full repertoire of important pre-existing antibody interactions against the capsid unless it has been uncoated, denatured, or digested. Thus this method has limitations on footprint prediction accuracy. For example, peptide scanning identified A20 epitope peptides that were adjacent to the footprint identified by cryo-reconstruction and model building but none overlapped (Table 2). In fact the peptides identified by scanning were mostly located under the capsid surface VRs that contained the epitope regions identified by site-directed mutagenesis and cryo-reconstruction. For C37-B, one of the peptides mapped by scanning, residues 493–502, included the residues on one of the amino acid stretches, 492–498, predicted to be within the footprint by the cryo-reconstruction (Table 2). The other peptide identified by peptide scanning, 601–610, is buried inside the capsid, and not predicted to interact with the antibody in the reconstructed complex structure. Residues 585–589 predicted to form part of the C37-B epitope by cryo-reconstruction was not identified by peptide scanning. The structural-based approach, either to confirm previously predicted epitopes, e.g., the AAV2:A20 and AAV2:C37-B complex structures, or to identify new epitopes, e.g., the AAV1:4E4, AAV1:5H7, and AAV8:ADK8 complex structures, is able to accurately map antibody footprints. However, to date, all the structures determined are for viruses complexed with Fabs generated from mouse MAbs. This is because cloning MAbs from human B-cells is challenging and structural studies with polyclonal human antibodies could lead to poorly resolved densities due to the variability in the sample. It could be argued that the murine immune response differs from that of humans and that human serum represents a polyclonal population of antibodies, and thus modification of single mouse MAb footprints mapped by cryo-reconstruction studies may not generate vectors that evade neutralization from human serum. To overcome this bottleneck and mimic the polyclonal response, the structure of each clinically relevant AAV complexed with Fabs from several MAbs can be determined to obtain information on dominant antigenic regions.

Given the advantages and disadvantages of the approaches described above, a two pronged-attack, concurrent directed evolution and structural mapping, is likely optimal for defining the capsid surface antigenic properties of AAVs. The available 3D structures for AAV1 to AAV9 provide the platform required for the visualization of epitopes obtained by directed evolution onto the parent serotypes. This can inform further modifications for desired tropisms, on the background of an escape mutant, given information on tropism determinants. Structural mapping will provide information that can enable rational engineering of vectors for escape mutation while retaining natural tropisms. Thus both strategies will delineate dominant epitopes for the AAVs. The current data, using both approaches, point to the protrusions around the icosahedral 3-fold axis and 2/5-fold wall, as the dominant targets for future modification for antibody escape.

Regardless of the method used to obtain the antigenic information, the ultimate goal is to create an AAV variant that can evade the neutralizing effect of a pre-existing immune response and has the capacity to effectively assemble genome packaged vectors and retain efficient cell tropism. The observations described in this review show that minor and local variations on the AAV capsid surface, including those due to single amino acid substitutions, may alter more than one phenotype of AAV vector, including tropism and antigenicity. The antigenic mapping data at hand, combined with efforts at chemical capsid modifications, pharmacological immuno-suppression, plasmapheresis, and saline flushing, point to potential strategies for improving the clinical efficacy of this promising gene delivery system.

## Conflict of Interest Statement

The authors declare that the research was conducted in the absence of any commercial or financial relationships that could be construed as a potential conflict of interest.
